# School Satisfaction and Happiness in 10-Year-Old Children from Seven European Countries

**DOI:** 10.3390/children8050370

**Published:** 2021-05-08

**Authors:** Diego Gómez-Baya, Francisco José García-Moro, Alicia Muñoz-Silva, Nuria Martín-Romero

**Affiliations:** 1 Department of Social, Developmental and Educational Psychology, University of Huelva, 21007 Huelva, Spain; fjose.garcia@dpsi.uhu.es (F.J.G.-M.); amsilva@dpsi.uhu.es (A.M.-S.); 2 Department of Educational Sciences, University of Alcala, 19001 Guadalajara, Spain; nuria.romerom@uah.es

**Keywords:** school satisfaction, happiness, subjective well-being, children, European countries

## Abstract

School satisfaction is conceptualized as a crucial factor influencing children´s happiness and consequent healthy functioning in multiple developmental areas. Research to date has mainly evaluated how contextual factors related to the interactions between the student, teachers and classmates influence children’s happiness, not considering other important factors more related to their own student experiences. The aim of the present study was to examine the effect of school satisfaction on happiness in 10-year-old children from Europe. Children’s global school satisfaction levels, as well as different separate indicators of school satisfaction (i.e., satisfaction with other children in class; school marks; school life experience as a student; things they have learned; and relationships with teachers) were considered. The study comprised a sample of 7.445 10-year-old children from seven European countries. First, correlation analysis showed that the overall school satisfaction measure, as well as its different indicators, had positive associations with happiness levels. Second, regression analyses confirmed the effect by indicators of global school satisfaction on happiness. The indicators with the strongest effects were the satisfaction with their life as a student and the satisfaction with other children in the class, while the smallest effects were found regarding the satisfaction with the relationships with teachers and the things learned. These results point out the need to consider personal and contextual indicators of school satisfaction in a program design to foster happiness in 10-year-old children.

## 1. Introduction

In the last years there has been an increasing number of international studies evaluating children’s well-being [1], due in part to the importance given to children’s well-being and its links to policy-relevant domains [2,3], especially regarding education [4]. In this way, a growing body of literature has shown how subjective psychological well-being, frequently assessed through measures of happiness, is related to school adjustment [5]. Subjective well-being refers to a person’s cognitive (i.e., life satisfaction) and affective evaluations (i.e., positive and negative affect) of his/her life [6]. As for school adjustment, it is a construct related to academic achievement, prosocial behaviors, positive relationships with peers and teachers [7,8], and to positive personal attributes (e.g., self-efficacy, self-concept, life satisfaction, and positive affect), that facilitate the student´s adaptation to school demands [9,10]. Therefore, school adjustment is considered a key factor for interventions aimed to face school failure and behavioral problems [11], as well as low academic expectations [12,13], and to prevent school dissatisfaction and school dropouts [14].

Importantly, the study of well-being and its role in school adjustment has comprised a substantial change in respect to a more traditional psychopathology-oriented approach, now focusing the attention on understanding and intervening in individuals’ competences and their positive development rather than on children’s disabilities [15,16,17]. This change of perspective has generated additional attention to non-academic, positive indicators of child well-being, including their perceptions of the overall quality of their lives and school experiences [18]. In this sense, school satisfaction (i.e., a student’s judgement of positivity of his or her school experience as a whole) [19] has been considered as an important contributing factor to overall life satisfaction [20]. Some indicators within school life have been identified as important in previous studies, including the quality of relationships between teachers and the student and between the student and school peers [18,21,22]. The results of these studies pointed out the importance of considering these contextual factors when aiming to promote school satisfaction in children. Beyond contextual factors related to teachers and peer relationships, there is a lack of studies considering other important factors concerning student experiences.

The importance of the school in a child’s happiness is also fundamental considering the time they spend in that institution, often being the main source of social interactions [4,23]. Thus, considering this, school satisfaction becomes an important construct to understand school adjustment and children’s quality of life. In this sense, several studies have reported the benefits of school satisfaction and positive school experiences in promoting perceived well-being and general life satisfaction. For instance, feeling satisfied at school and having school success both contribute to a general life satisfaction [20,24]. Moreover, the presence of positive role models at school significantly contributes to the development of students’ competences and positive development [17,22,25,26]. Also, positive relationships have been found between school adaptation and perceived well-being [27]. Finally, children with higher life satisfaction scores show, in turn, higher scores on all measures of academic functioning [28,29]. 

Overall, school is one of the main contexts of human development and therefore the principal place for facilitating and promoting happiness for children and young people. However, applying positive psychology at school requires rethinking our concept of education, considering happiness or subjective well-being within the objectives of the educational plan, and introducing changes in school organization and teaching methods [30,31]. This has the potential to favor an adequate classroom climate, as well as optimal learning and reduction of behaviors that could make difficult the pursuit of this end [32]. For this reason, it is first important to establish children’s happiness levels, according to their self-perceptions [33,34], but, second, it is further essential to know how to intervene in the elements that generate this set of perceptions, such as the school climate, the role of teachers, peer group dynamics in the classroom, the curriculum design, or the academic results. The goal is thus not only preventing risk behaviors but promoting positive functioning in young people, since health goes beyond the absence of disease, and therefore does not guarantee a positive development in school and society [35,36]. It is necessary to carry out concrete actions to promote healthy conditions through the development of skills, behaviors, and competences that are necessary to be successful in social, academic, and professional life [35,36].

Although there is growing literature evaluating subjective well-being or happiness in children and the factors that could affect its development, such as school satisfaction, few studies have assessed if these associations vary across different countries. In this sense, a recent study evaluated the associations of children’s well-being with different factors such as family structure, children’s relationships with their families, friends and school, in four European countries [37]. Results showed that family structure and social relationships regarding family, friends and teachers, all significantly correlated and predicted children’s well-being. Previously, Klocke et al. [38] and Holder and Coleman [39] both found that family and school relationships explained children’s subjective well-being across different countries. Similarly, Bradshaw, Martorano, Natali, and de Neubourg [40] found that the relationships with classmates, the pressures of work, and school satisfaction levels, all predicted children’s well-being. Finally, Lee and Yoo [41] found that school life, measured by frequency of peer activity, experience of bullying, and school safety, were all significant predictors of subjective well-being even after controlling for country-specific cultural and contextual factors.

The objective of the present study was to extend this initial evidence, evaluating how different global and specific dimensions of school satisfaction referred to both contextual and students’ personal experience factors, explain 10-year-old children’s happiness levels across seven European countries. More specifically, we wanted to evaluate how global school satisfaction and their different specific indicators (i.e., satisfaction with other children in his/her class; with student school marks; with the school experience of his/her life as a student; with things he/she had learned; and with his/her relationships with teachers) have an effect on individual levels of happiness. Taken into account previous studies [18,21,22], we hypothesized that global school satisfaction and its different indicators would be related to higher levels of happiness in 10-year-old children in all seven European countries evaluated in the study. 

## 2. Materials and Methods

### 2.1. Data Collection Procedure

Data were extracted from the second wave of the Children’s Worlds project, an international survey of children’s lives and well-being [42]. In this study, a database of 10-year-old children from seven European countries (i.e., Estonia, Spain, Germany, Romania, Norway, Poland, and Malta) was used. Its objective was to promote the comparative study between countries based on the data provided by the children themselves about their perceptions and evaluations of their lives in their different environments, mainly school and family. The data are freely available at the website www.isciweb.org (accessed on 1 April 2021) for all interested researchers for academic purpose and by request. The authors of this manuscript completed the request of the data and received the authorization. The present manuscript used the database from the second wave because it collected the most recent available data about 10-year-old children in this project. Using these secondary data with permission, only data from the seven European countries included in the overall international study were examined in order to provide an image of this concrete continent. 

The design of this study was cross-sectional and descriptive, and data were collected during the winter of 2013 and the spring of 2014. A representative sample of the participating countries filled in a self-reporting questionnaire during school time. Ethical approval from the appropriate ethics boards in each country was obtained, and parents’ and children’s informed consents were collected. The data collection respected confidentiality, privacy, and anonymity of participants. More detailed information on subject recruitment and sampling procedure by country is presented in the reports published on the web at: https://isciweb.org/the-data/publications/country-reports/country-reports-of-the-second-wave-2013-2014/ (accessed on 1 April 2021).

### 2.2. Participants

In the present work, the total sample was composed of 7445 children (47.9% girls, 51.6% boys, and 0.5% missing), aged 10 years old, from Estonia, Spain, Germany, Romania, Norway, Poland, and Malta. Table 1 shows descriptive statistics of the sample by gender and country. In the overall sample, 94.4% of the sample was born in the country where they lived. Each country followed a sampling procedure to reach a representative sample of around 1000 children surveyed in the project. All participants were enrolled at a school chosen for the study. They provided informed consent and attended class on the day of the fieldwork.

### 2.3. Instrument

A back-translation procedure was followed from English to the native language in each country. The questionnaire was previously piloted to examine psychometric properties [43]. For the reasons of the present research, only demographics (i.e., gender, age, and nationality), school satisfaction items, and happiness indicator were used.

Happiness. This variable was assessed with the question “Overall, how happy have you been feeling during the last two weeks?”, with response options from 0 “Not at all happy” to 10 “Totally happy”. 

Satisfaction with school. The scale comprised six indicators, introduced by a question regarding the level of satisfaction: “How satisfied are you with each of the following things in your life?: Other children in your class?; Your school marks?; Your school experience?; Your life as a student?; Things you have learned?; and Your relationship with teachers?”. The response options were coded from 0 “Not at all satisfied” to 10 “Totally satisfied”. Notable internal consistency reliability was observed, with α = 0.83. Good factorial validity was observed, χ2(9) = 239.67, *p* < 0.001, CFI = 0.983, NNFI = 0.972, SRMR = 0.022, RMSEA = 0.062, and 90% CI RMSEA = 0.055 – 0.069. All measurement equations were significant and standardized and residuals were very low. Table 2 presents factor loadings of the indicators of school satisfaction, the Average Variance Extraction (AVE) and Composite Reliability (CR). Factor loading were all over 0.60, AVE was higher than 0.50, and CR was over 0.70. Thus, the scale showed good psychometric properties, both reliability, factor validity, and discriminant validity.

### 2.4. Data Analysis Design

First, the descriptive statistics (i.e., mean and standard deviation) of happiness and the separate items of school satisfaction were examined in the total sample and by country. As well, a mean score was calculated for school satisfaction based on its respective indicators. Second, bivariate Pearson zero-order correlations were calculated between school satisfaction items and happiness in the total sample. Third, stepwise regression analyses were conducted to analyze happiness based on the demographics in step 1 (i.e., gender and nationality) and separate school satisfaction indicators in step 2. F, R2, and standardized coefficients (β) were reported in the total sample and by country. Multicollinearity was examined, as well as self-correlation (Durbin–Watson test). These analyses were performed with JASP 0.14.1 and SPSS 21.0. 

## 3. Results

### 3.1. Descriptive Statistics

Table 3 presents descriptive statistics (mean and standard deviation) of happiness and school satisfaction (both overall score and separate indicators) in the total sample and by country. Notable scores were observed in happiness and school satisfaction. Higher means in school satisfaction were found in the satisfaction with the things learned and with the relationships with teachers, while the lower ones were detected in the satisfaction with other children in class and with school marks. 

Comparing the scores in the different countries, higher means in happiness were found in Romania and Spain, while less happiness was reported in Estonia and Germany. Regarding school satisfaction, greater scores were observed in Romania and Norway, while lower scores were found in Germany and Estonia. Significant differences between countries were found in school satisfaction, F(6, 6707) = 54.24, and *p* < 0.001, η^2^_p_ = 0.05, and in happiness, F(6, 7309) = 55.90, and *p* < 0.001, η^2^_p_ = 0.04. Girls (M = 8.89, SD = 1.29) reported more school satisfaction than boys (M = 8.66, SD = 1.52), t(6683) = –6.58, *p* < 0.001, and md = –0.23. No gender differences were found in happiness, t(7281) = –0.49, and *p* = 0.622. Concerning nationality, children who were born in the country they lived in showed more school satisfaction (M = 8.78, and SD = 1.41) than those who were born in another country (M = 8.60, and SD = 1.54), t(6683) = –2.33, *p* = 0.020, and md = –0.18. No differences in nationality were found in happiness, t(7285) = –1.80, and *p* = 0.072.

### 3.2. Bivariate Correlations

Table 4 shows bivariate correlations between school satisfaction and happiness in the overall sample. Positive interrelations were found between school satisfaction indicators and happiness in the overall sample. Moderate to large correlation was observed between overall school satisfaction and happiness. The strongest associations with happiness were presented by satisfaction with the life as student, the school experience, and other children in class. The lowest associations were observed with the relationships with teachers. Furthermore, large positive interrelations were detected between the indicators of school satisfaction. The strongest associations were found between satisfaction with school experience and with life as a student, and between the satisfaction with the things learned and the life as a student.

### 3.3. Hierarchical Regression Analyses

Table 5 presents the results of the hierarchical regression analyses to explain child happiness based on demographics and school satisfaction indicators. In the first step, country differences were observed in happiness, as pointed out above. No remarkable effects were observed by gender or nationality, presenting no significant levels of explained variance. In the second step, school satisfaction indicators were introduced and presented positive effects on happiness, reaching 22% of explained variance. The indicators with the strongest effects were the satisfaction with life as a student, and the satisfaction with other children in the class, while the smallest effects were found regarding the satisfaction with relationships with teachers, and the things learned. 

Some differences between the participating countries were detected. The strongest effect by the satisfaction with other children in the class was observed in Spain, Poland, and Romania. The effect of school marks was especially high in Estonia, while school experience was more remarkable in Romania. In Norway and Germany, the satisfaction with life as a student was stronger than in other countries. In Germany and Estonia, the satisfaction with the things learned had a positive effect on happiness, while no effect was observed in other countries. Only in Malta the satisfaction with relationships with teachers had a positive effect on happiness. Concerning R–squared values, the countries in which school satisfaction indicators presented more effect on child happiness were Norway, Estonia, Germany, and Poland, with lower scores in Malta, Romania, and Spain.

## 4. Discussion

The aim of the present study was to evaluate the effect of school satisfaction on happiness in 10-year-old children from seven European countries (i.e., Estonia, Spain, Germany, Romania, Norway, Poland, and Malta). More specifically, we aimed to establish how different factors regarding school satisfaction, including contextual and students’ own experience factors (i.e., satisfaction with other children in the class; student school marks; the student’s school life experience; things learned; and relationships with teachers), predicted individual differences in children’s happiness. 

First, our study provides evidence of high scores in happiness and school satisfaction in the total sample. Higher means in school satisfaction were particularly found in satisfaction with the things learned and with relationships with teachers. In contrast, the lower means were detected in the measures of satisfaction with other children in the class and with school marks. These results point out that children from the seven countries evaluated were more satisfied with those factors that were inherent to the school context (i.e., things learned, and relationship with teachers), and less satisfied with those factors of a more individual nature and those related to peer relationships (i.e., school marks, and other children in the class). These results are in line with those obtained by Arciuli, Emerson and Llewellyn [44], who found that school satisfaction was primarily related to satisfaction with teachers’ support, and, to a much lesser extent, parental support and the number of close friendships. And also, with those obtained by the study of Baker [45], who suggested that students’ perception of a caring and supportive school community had the most substantive impact of the variables considered on their satisfaction with school.

Regarding country comparisons, descriptive analyses showed significant differences between countries in school satisfaction and happiness. We found a clear pattern in which Romania showed the highest scores in both school satisfaction and happiness, while Germany and Estonia showed the worst scores in both measures. Interestingly, a previous study also found that Germany showed the lowest level of happiness compared to the rest of the countries evaluated (i.e., Spain, Norway, Germany, and the United Kingdom). Taking into account how education policies can influence students’ happiness [4], it would be interesting to analyze the structure of the education systems followed by the different countries in a more detailed manner in order to clarify the meaning of these differences. 

Our results also showed gender differences regarding school satisfaction, with girls being more satisfied than boys with schooling. However, no gender differences were found in happiness. Previous studies have found gender differences in subjective well-being, showing that girls have lower subjective well-being than boys [37,38,41]. However, it is important to take into account the different measures used across studies to evaluate happiness or subjective well-being (i.e., one-item measures vs. multiple-item measures). Further, in our study, the sample comprised 10-year-old children, while in the rest of the previous studies mentioned above the samples were more heterogeneous in age rank, also including adolescents. A previous large-scale study including European Union countries, the United States of America, and Canada has shown how girls’ happiness declines with age [46], being similar between boys and girls aged 11, but then later decreasing specifically in girls. Similar to our results, Uusitalo-Malmivaara [47] found no gender differences in happiness when considering children from the sixth grade. 

Correlation analyses showed positive relations between school satisfaction indicators and happiness levels in the overall sample, as well as between the different indicators of school satisfaction and happiness. The strongest associations with happiness were found for satisfaction with life as a student, with the school experience, and with other children in the class. The lowest associations were found for satisfaction with relationships with other children. Further hierarchical regression analyses revealed that the indicators with strongest predictive effects in happiness levels were the satisfaction with the life as a student and the satisfaction with other children in the class, while the smallest effects were found for the satisfaction with relationships with teachers and with the things learned. These results show that happiness seems to be more influenced by individual indicators referred to satisfaction with a student’s life as well as with peer relationships, the latter also supported in previous studies [18,21,47]. Therefore, satisfaction with a student’s life and satisfaction with other children in the class seem to be the most important factors in which to intervene, in order to promote happiness in 10-year-old children. 

Hierarchical regression analyses conducted by countries showed that the strongest effect of satisfaction with other children in the class was observed in Spain, Poland, and Romania. The effect of satisfaction with school marks was especially high in Estonia, while satisfaction with the school experience had a stronger effect in Romania. In Norway and Germany, satisfaction with life as a student had the strongest effect in children’s happiness. Further, in Germany and Estonia, the satisfaction with the things learned had a positive effect on happiness, while this effect was not observed in other countries. Finally, only in Malta, satisfaction with relationships with the teachers had a positive predictive effect on happiness. All these results show a large heterogeneity when considering what school satisfaction factors can influence children’s happiness, highlighting the relevance of different school satisfaction factors for different countries. Future research should aim to analyze the educational systems and student relational patterns in each country in order to clarify the meaning of these differences [4]. For instance, in her study, Moreno [37] found that the children’s perception about time spent with their parents contributes to enhanced happiness in Germany and Norway, but not in Spain and the United Kingdom. This author argued that highly developed work–family balance policies in Germany and Norway compared with less developed policies in Spain and the United Kingdom could explain these observed differences. More extensive work in this area, regarding factors accounting for differential roles of school satisfaction factors in children’s happiness, promises to improve our understanding of new avenues to promote school adjustment and healthy development.

Consistent with previous studies, analyses by country showed that more school satisfaction was related with more child happiness in the six European countries examined [20,24,27,48]. More effects on these relationships were found in Estonia, Germany and Norway, while fewer effects were observed in Romania and Malta. These results are interesting and somewhat surprising considering that Estonia, together with Germany, was the country that showed less school satisfaction and less happiness. In contrast, Romania, followed by Spain and Norway, were the countries that showed more school satisfaction and more happiness. These results point out that, although Estonia reported less school satisfaction and less happiness, these relationships became stronger compared to the other countries evaluated in the study. Furthermore, although Romania reported more school satisfaction and more happiness, these relationships were weaker than in the other countries. It is possible that school satisfaction has more or less weight on children’s levels of happiness depending on the country, being more important in Estonia than in Romania. As seen in previous studies, differences in social domains and educational policies may explain the nature of these relationships [4,37].

In relation to practical implications, it is important to consider that happiness is beneficial in different life domains, including friendship and health [49]. In that way, there is evidence that happy people tend to reflect on good things that have happened in the past, focus on how life problems can be fixed, and tend to the belief that life is under their control compared with unhappy people [50]. Programs aimed at increasing happiness should begin during childhood. Emotional, personality, and social development occurring during this time period make it the most opportune time for change to occur and to create a strong basis for positive well-being throughout life [51]. Based on our results supporting that school satisfaction predicts happiness in 10-year-old children, programs promoting school satisfaction can be very beneficial.

Based on previous evidence regarding the utility of intervention programs based on positive psychology in the school context [52], we highlight the need to implement this type of programs to promote happiness and life satisfaction in children. Some authors, such as Cleveland and Sink [53], defend the inclusion of subjective life satisfaction or happiness as part of the evaluation of the school climate in school improvement plans. In the same way, authors focusing on the paradigm of Resilience Education [54] point out that schools have the enormous potential and responsibility to promote resilience and well-being in children. Moreover, Positive Education includes the instruction of both traditional skills and happiness [54]. Following this Positive Education paradigm, White [55] summarizes the scientifically validated programs of positive psychology taught in schools with a demonstrated impact on the well-being of students. We argue that our findings highlight the relevance of integrating these approaches in European schools and that activities aimed at improving different factors of school satisfaction can be central to promoting students’ well-being and, consequently, promoting adaptive functioning at school and achieving healthy developmental outcomes.

The present study has some strengths and limitations. Regarding the strengths, our study comprised a large sample of 7445 children from seven different European countries. Moreover, we considered different indicators of school satisfaction. In that way, as in previous studies, we evaluated the students’ perceptions regarding relationships with classmates and teachers [37]. But, further, in our study we also included other series of indicators more related with students’ personal experiences, such as students’ school life experience, school marks, and things learned. Finally, it is important to highlight the consideration of the students’ own experiences and perceptions, which has been considered as a fundamental aspect of school satisfaction by several authors [33,34,56]. With respect to the limitations, it is important to take into account the cross-sectional nature of our study, which precludes making assumptions of clear causal relationships between school satisfaction and happiness. Future longitudinal studies may consider the possibility of using different measures of school satisfaction and happiness over time in order to fully establish the causal relationships between the proposed predictor variables (i.e., school satisfaction indicators) and the outcome variable (i.e., happiness). The selection of boys and girls of a specific age—10 years—is also a limitation of this study, which could be improved in future research by examining data from different ages across adolescence. As well, the use of single-item measures to assess happiness may limit the conclusions on this construct and more complete measures are recommended in future research.

## 5. Conclusions

In summary, our study provides evidence about the positive relationship between school satisfaction factors and happiness in 10-year–-old children from seven European countries. Interestingly, going beyond general global measures of school satisfaction, we used a dataset that allowed disentangling different school satisfaction indicators which showed differential associations with happiness levels across the countries evaluated. Regression analyses showed how satisfaction with student life and satisfaction with other children in the class were the variables with stronger effects on the whole sample. These results point out the need to consider personal and contextual factors related to school satisfaction, especially those related to a student’s life and to relationships with peers, in order to promote happiness in 10-year-old children.

## Figures and Tables

**Table 1:** Descriptive statistics of the sample by country and gender.

Country	Gender	Frequency	%	Valid %	Cumulative %
Estonia	Boy	520	51.33	51.84	51.84
	Girl	483	47.68	48.16	100.000
	Missing	10	0.99		
	Total	1013	100.000		
Spain	Boy	544	51.47	51.47	51.47
	Girl	513	48.53	48.53	100.00
	Missing	0	0.000		
	Total	1057	100.000		
Germany	Boy	532	48.32	48.63	48.63
	Girl	562	51.04	51.37	100.000
	Missing	7	0.64		
	Total	1101	100.000		
Romania	Boy	709	52.32	52.71	52.71
	Girl	636	46.94	47.29	100.00
	Missing	10	0.74		
	Total	1355	100.00		
Norway	Boy	473	49.27	49.43	49.43
	Girl	484	50.42	50.57	100.00
	Missing	3	0.31		
	Total	960	100.00		
Poland	Boy	585	52.28	52.47	52.47
	Girl	530	47.36	47.53	100.00
	Missing	4	0.36		
	Total	1119	100.00		
Malta	Boy	480	57.14	57.14	57.14
	Girl	360	42.86	42.86	100.00
	Missing	0	0.000		
	Total	840	100.00		
TOTAL	Boy	3843	51.62	51.86	51.86
	Girl	3568	47.92	48.14	100.000
	Missing	34	0.46		
	Total	7445	100.00		

**Table 2:** Factor loadings of the indicators of school satisfaction, Average Variance Extraction and Composite Reliability.

	Average Variance Extraction	Concept Reliability	Factor Loading
Overall scale	0.55	0.88	
Other children in your class			0.64
Your school marks			0.67
Your school experience			0.80
Your life as a student			0.82
Things you have learned			0.78
Your relationship with teachers			0.74
Overall school satisfaction			0.64

**Table 3:** Descriptive statistics of happiness and school satisfaction in the total sample and by country.

	Total	Estonia	Spain	Germany	Romania	Norway	Poland	Malta
	M(SD)	M(SD)	M(SD)	M(SD)	M(SD)	M(SD)	M(SD)	M(SD)
Happiness	8.77(1.88)	8.16(2.14)	9.02(1.63)	8.30(2.08)	9.34(1.44)	8.88(1.86)	8.86(1.65)	8.66(2.16)
Other children in your class	8.47(1.96)	8.11(2.15)	8.60(1.92)	8.05(1.95)	8.67(1.93)	8.92(1.69)	8.38(2.00)	8.60(1.93)
Your school marks	8.47(2.08)	8.19(2.22)	8.34(2.01)	7.97(2.11)	8.96(1.97)	8.84(1.85)	8.38(2.09)	8.52(2.10)
Your school experience	8.79(1.89)	8.69(2.07)	8.80(1.83)	8.34(1.98)	9.24(1.53)	8.98(1.77)	8.59(2.00)	8.79(1.98)
Your life as a student	8.76(2.01)	8.52(2.19)	8.63(1.91)	8.38(2.08)	9.42(1.52)	8.96(1.90)	8.42(2.18)	8.84(2.08)
Things you have learned	9.14(1.63)	8.89(1.83)	9.23(1.41)	8.90(1.55)	9.57(1.24)	9.18(1.60)	8.84(1.97)	9.29(1.55)
Your relationship with teachers	8.86(2.00)	8.51(2.28)	8.88(1.91)	8.54(1.90)	9.29(1.71)	9.32(1.60)	8.40(2.37)	9.08(1.85)
Overall school satisfaction	8.76(1.42)	8.50(1.60)	8.72(1.33)	8.36(1.45)	9.22(1.08)	9.04(1.33)	8.54(1.60)	8.88(1.29)

**Table 4:** Bivariate correlations between school satisfaction and happiness in the overall sample.

	1	2	3	4	5	6	7	8
1. Happiness	1							
2. Other children in your class	0.35 ***	1						
3. Your school marks	0.30 ***	0.32 ***	1					
4. Your school experience	0.35 ***	0.46 ***	0.46 ***	1				
5. Your life as a student	0.39 ***	0.43 ***	0.47 ***	0.61 ***	1			
6. Things you have learned	0.31 ***	0.36 ***	0.43 ***	0.51 ***	0.59 ***	1		
7. Your relationship with teachers	0.28 ***	0.35 ***	0.39 ***	0.47 ***	0.52 ***	0.55 ***	1	
8. Overall school satisfaction	0.45 ***	0.66 ***	0.69 ***	0.79 ***	0.81 ***	0.76 ***	0.74 ***	1

*** *p* < 0.001.

**Table 5:** Hierarchical regression analyses to explain child happiness based on demographics and school satisfaction indicators in the total sample and by country.

		Total	Estonia	Spain	Germany	Romania	Norway	Poland	Malta
Step 1	F R^2^	21.23 *** 0.01	2.03 0.01	0.62 0.01	0.60 0.01	2.40 0.01	1.96 0.01	1.37 0.01	0.18 0.01
Country	β	0.10 ***							
Gender	β	0.01	0.07 *	–0.01	–0.02	0.05	–0.02	0.03	–0.02
Nationality	β	0.02	–0.01	0.04	0.03	0.03	0.06	0.04	0.01
Step 2	F R^2^	200.49 *** 0.22	38.15 *** 0.24	16.69 *** 0.15	38.31 *** 0.24	25.61 *** 0.14	37.77 *** 0.25	40.39 *** 0.23	17.26 *** 0.14
Country	β	0.05 ***							
Gender	β	–0.02	0.01	–0.04	–0.04	0.03	–0.05	0.02	–0.04
Nationality	β	0.01	–0.01	–0.01	–0.01	0.03	0.04	0.01	0.01
Other children in your class	β	0.17 ***	0.15 ***	0.22 ***	0.13 ***	0.20 ***	0.13 ***	0.21 ***	0.14 ***
Your school marks	β	0.09 ***	0.17 ***	0.02	0.09 **	0.01	0.11 **	0.12 **	0.10 **
Your school experience	β	0.09 ***	0.10 *	0.08	0.03	0.15 ***	0.08	0.11 **	0.06
Your life as a student	β	0.18 ***	0.11 **	0.15 **	0.25 ***	0.14 ***	0.27 ***	0.17 ***	0.16 ***
Things you have learned	β	0.05 **	0.08 *	0.03	0.10 *	0.03	–0.01	–0.03	0.03
Your relationship with teachers	β	0.03 *	0.05	0.04	0.03	–0.04	0.04	0.04	0.09 *

*** *p* < 0.001, ** *p* < 0.01, and * *p* < 0.05.

## Data Availability

The data used in this publication correspond to the second wave of Children’s Worlds project: An international survey of children’s lives and well-being (www.isciweb.org (accessed on 1 April 2021)). The views expressed in this manuscript are those ones of the author(s). They are not necessarily those of the authors of the ISCWeB project.
